# Increased brain gyrification and cortical thinning in winter-born patients with schizophrenia spectrum

**DOI:** 10.3389/fpsyt.2024.1368681

**Published:** 2024-04-24

**Authors:** Misako Torigoe, Tsutomu Takahashi, Yoichiro Takayanagi, Daiki Sasabayahi, Haruko Kobayashi, Kazumi Sakamoto, Yusuke Yuasa, Noa Tsujii, Kyo Noguchi, Michio Suzuki

**Affiliations:** ^1^ Department of Neuropsychiatry, University of Toyama Graduate School of Medicine and Pharmaceutical Sciences, Toyama, Japan; ^2^ Research Center for Idling Brain Science, University of Toyama, Toyama, Japan; ^3^ Arisawabashi Hospital, Toyama, Japan; ^4^ Department of Child Mental Health and Development, Toyama University Hospital, Toyama, Japan; ^5^ Department of Radiology, University of Toyama Graduate School of Medicine and Pharmaceutical Science, Toyama, Japan

**Keywords:** birth season, gyrification, neurodevelopment, schizophrenia, magnetic resonance imaging

## Abstract

**Introduction:**

The findings of epidemiological studies suggest that a relationship exists between the risk of schizophrenia and winter births in the Northern Hemisphere, which may affect the process of fetal neurodevelopment. However, it remains unclear whether birth seasons are associated with the brain morphological characteristics of patients within the schizophrenia spectrum.

**Methods:**

The present magnetic resonance imaging study using FreeSurfer software examined the effects of birth seasons (i.e., summer-born *vs.* winter-born) on the comprehensive brain surface characteristics of 101 patients with schizophrenia (48 summer- and 53 winter-born), 46 with schizotypal disorder (20 summer- and 26 winter-born), and 76 healthy control subjects (28 summer- and 48 winter-born).

**Results:**

In comparisons with summer-born patients, winter-born patients, particularly those with schizophrenia, showed significantly increased gyrification mainly in the left lateral occipital and inferior temporal regions and right fronto-parietal region as well as cortical thinning in the right superior frontal region. Birth seasons did not significantly affect the local gyrification index or cortical thickness in healthy controls.

**Discussion:**

The present whole-brain surface-based analysis demonstrated that brain morphological characteristics reported in the schizophrenia spectrum were more pronounced in winter-born patients than in summer-born patients, suggesting the contribution of early neurodevelopmental factors associated with birth seasons to the pathophysiology of the schizophrenia spectrum.

## Introduction

Evidence from epidemiological studies in Northern Hemisphere countries has consistently suggested that a relationship exists between birth seasons and the risk of mental disorders (1), with the risk of developing schizophrenia (Sz) and related disorders being approximately 5-10% higher for winter/spring births (2, 3). Vitamin D deficiency or gestational exposure to infection and the associated inflammatory response during winter may increase susceptibility to Sz by affecting prenatal neurodevelopment, such as synapse formation and interneuron migration (4–6). However, previous magnetic resonance imaging (MRI) studies failed to detect a significant effect of birth seasons on ventricular (3) or hippocampal (7) volumes or various neurodevelopmental markers (e.g., gross gyral patterns in the orbitofrontal and insular regions) (8) that are characteristic of Sz. Nevertheless, a more comprehensive brain assessment, particularly of brain characteristics closely reflecting early neurodevelopment, such as whole-brain cortical gyrification patterns (9, 10), may provide more detailed insights into the effects of birth seasons on the brain morphology of patients with the Sz spectrum.

MRI studies on Sz and schizotypal (SzTypal) disorder, often described as a milder form within the Sz spectrum (11, 12), revealed shared brain characteristics, such as increased gyrification in the fronto- parietal regions (13) and a reduction in cortical thickness in the medial temporal and frontal regions (14), potentially reflecting a common vulnerability to psychopathology. While the cortical volume or thickness may be significantly affected by various factors after birth (e.g., antipsychotic medication and an active brain pathology during the early stages of Sz) (15), the cortical gyrification pattern is considered to mainly be formed during gestation and remains stable after birth (16, 17). Therefore, changes in cortical gyrification commonly observed in the Sz spectrum may more closely reflect an early neurodevelopmental pathology (including the disruption of neural development associated with seasonality) (10). While human cortical folding is likely to be more genetically mediated compared to other surface characteristics (e.g., curvature, cortical thickness or volume, and surface area), higher order sulci formed during late embryonic period are reported to be less heritable and more influenced by environmental factors than ontologically older sulci (18). However, to the best of our knowledge, the potential effects of birth seasons on the comprehensive surface morphology in patients with the Sz spectrum have not yet been examined using MRI.

Therefore, the present MRI study aimed to expand our preliminary negative study of selected brain regions (8) by using whole-brain cortical surface morphometrics, particularly the local gyrification index (LGI). We predicted that increased brain gyrification in the Sz spectrum (13) would be more pronounced in winter-born patients than in summer-born patients due to potential adverse effects associated with a winter birth on neurodevelopment *in utero* (4, 6). We also examined cortical thickness, a representative parameter of focal gray matter changes among the parameters in the surface-based approach (e.g., volume, area) (14, 19), but predicted a weaker relationship with respect to birth seasons due to various potential confounding factors during the course of illness.

## Materials and methods

### Participants

One hundred and one patients with Sz, 46 with SzTypal disorder, and 76 healthy control (HC) subjects were included in the present study (**Table 1**). Participants in the present study were all born in Japan (i.e., the Northern Hemisphere), right-handed, physically healthy at the time of the study, and had no history of severe obstetric complications, serious head trauma, neurological illness, serious medical or surgical illness, or substance abuse. While most of the participants were subjects in our previous studies on LGI (13) and cortical thickness (14) in the Sz spectrum, we herein examined the specific effects of birth seasons on these cortical measures for the first time; with reference to previous studies (7, 8, 20), participants born between June and October and between November and May were defined as summer-born and winter-born, respectively.

**Table 1:** Characteristics of study participants.

Season of birth	SzTypal (*N* = 46)		Sz (*N* = 101)		HC (*N* = 76)			
	Summer (*N* = 20)	Winter (*N* = 26)	Summer (*N* = 48)	Winter (*N* = 53)	Summer (*N* = 28)	Winter (*N* = 48)	Effect of diagnosis	Effect of season
Male/female	14/6	15/11	28/20	27/26	17/11	26/22	Chi-square = 0.96, *p* = 0.620	Chi-square = 1.40, *p* = 0.237
Age (years)	26.1 (5.4)	24.2 (5.5)	26.0 (5.8)	25.2 (5.3)	25.5 (6.0)	23.6 (5.4)	*F* (2, 217) = 0.75, *p* = 0.476	*F* (1, 217) = 3.64, *p* = 0.058
Height (cm)	168.0 (8.70)	164.8 (8.6)	165.6 (7.3)	164.0 (8.6)	167.9 (8.5)	166.1 (7.3)	*F* (2, 217) = 1.70, *p* = 0.185	*F* (1, 217) = 3.60, *p* = 0.059
Weight (kg)	64.0 (11.8)	60.2 (11.3)	60.2 (12.5)	59.9 (10.8)	59.2 (10.3)	58.8 (10.1)	*F* (2, 217) = 1.08, *p* = 0.343	*F* (1, 217) = 0.83, *p* = 0.362
Education (years)	13.1 (1.9)	13.0 (2.1)	13.3 (2.0)	13.6 (1.8)	16.6 (2.2)	15.7 (2.7)	*F* (2, 217) = 40.60, *p* < 0.001; SzTypal, Sz < HC	*F* (1, 217) = 0.48, *p* = 0.489
Parental education (years)^a^	12.2 (2.1)	12.5 (1.4)	12.4 (2.0)	12.5 (2.1)	12.8 (2.4)	12.9 (2.3)	*F* (2, 208) = 0.96, *p* = 0.385	*F* (1, 208) = 0.42, *p* = 0.517
Onset age (years)	–	–	22.6 (4.9)	21.5 (4.2)	–	–	–	*F* (1, 99) = 1.50, *p* = 0.224
Illness duration (months)	–	–	40.6 (52.8)	45.3 (59.2)	–	–	–	*F* (1, 99) = 0.18, *p* = 0.677
Medication dose (haloperidol-equivalent, mg/day)	5.5 (6.5)	4.4 (5.3)	10.6 (9.3)	10.3 (8.7)	–	–	*F* (1, 143) = 14.38, *p* < 0.001; SzTypal < Sz	*F* (1, 143) = 0.24, *p* = 0.622
Duration of medication (months)	28.8 (49.5)	11.0 (19.0)	30.8 (47.2)	31.0 (47.0)	–	–	*F* (1, 143) = 1.97, *p* = 0.163	*F* (1, 143) = 1.27, *p* = 0.262
Total SAPS scores^b^	17.4 (9.8)	15.0 (8.9)	24.3 (17.8)	31.3 (24.1)	–	–	*F* (1, 135) = 11.90, *p* < 0.001; SzTypal < Sz	*F* (1, 135) = 0.48, *p* = 0.491
Total SANS scores^b^	42.8 (21.7)	39.6 (21.2)	48.8 (21.5)	49.4 (25.5)	–	–	*F* (1, 135) = 3.58, *p* = 0.061	*F* (1, 135) = 0.09, *p* = 0.764
Intracranial volume (cm3)	1548.0 (127.1)	1507.0 (164.8)	1504.0 (159.3)	1489.7 (151.7)	1529.5 (146.0)	1472.6 (141.5)	*F* (2, 217) = 0.68, *p* = 0.507	*F* (1, 217) = 3.02, *p* = 0.084

Values represent means (SD) unless otherwise stated.

HC, healthy controls; SANS, scale for the assessment of negative symptoms; SAPS, scale for the assessment of positive symptoms; Sz, schizophrenia; SzTypal, schizotypal disorder.

a Data not available for one control, four SzTypal patients, and four Sz patients.

b Data not available for two SzTypal and six Sz patients.

As fully described elsewhere for inclusion/exclusion criteria (21–23), Sz and SzTypal disorder patients who met the ICD-10 research criteria (24) were enrolled from the Department of Neuropsychiatry, Toyama University Hospital. Patients underwent a structured interview by experienced psychiatrists using the Comprehensive Assessment of Symptoms and History (25), based upon which they were diagnosed with Sz and SzTypal disorder. Clinical symptoms were assessed with the Scale for the Assessment of Negative Symptoms and the Scale for the Assessment of Positive Symptoms (SANS/SAPS) (26). All SzTypal patients also fulfilled the Diagnostic and Statistical Manual of Mental Disorders (DSM) criteria for SzTypal personality disorder on the basis of the Structured Clinical Interview for DSM-IV Axis II Disorders (27); they had been clinically followed up for at least 2 years and had not developed overt psychosis. According to the literature (28–30), the first-episode Sz subgroup was defined by an illness duration ≤ 1 year (*n* = 48) or first psychiatric hospitalization (*n* = 16). Medication and other clinical data on patients are summarized in **Table 1**.

HC subjects from members of the community, staff at our hospital, and university students were screened using a questionnaire (31) on their family and personal medical histories. They did not have any personal or family history of psychiatric illnesses in their first-degree relatives. The control subjects of the present study were selected from our database with similar age and sex ratios to the patient groups. The present study was approved by the Committee on Medical Ethics of Toyama University (ID: I2013006). According to the guidelines of the Declaration of Helsinki, written informed consent was obtained from all participants after a full explanation of the protocol used in this study. A parent or guardian provided their written consent when the participant was younger than 20 years.

### Imaging and processing procedures

MRI was performed with a 1.5-T scanner (Magnetom Vision, Siemens Medical System, Inc., Erlangen, Germany) using a 3D gradient-echo sequence fast low-angle shots (FLASH) sequence providing 160-180 contiguous 1.0-mm-thick T1-weighted slices in the sagittal plane. Imaging parameters were as follows: repetition time/echo time = 24/5 ms, flip = 40°, matrix = 256×256 pixels, field of view = 256mm, and voxel size = 1.0 × 1.0 × 1.0 mm^3^.

FreeSurfer software (version 5.3; https://surfer.nmr.mgh.harvard.edu/) was used to process T1-weighted MR images according to a standard automatic reconstruction algorithm. The surface-based pipeline involved the following: removing non-brain tissue, a transformation to Talairach-like space, gray/white matter tissue segmentation, triangular tessellation, and inspections of white matter and pial surfaces (19). Following the careful inspection of each reconstructed image, the editing of segmentation errors was manually performed by a trained researcher (DS) blinded to the identities of participants. Since the aim of this study was to expand on our previous FreeSurfer findings (13, 14) using the same dataset/settings to avoid the confounding effects of different FreeSurfer versions (32, 33), we consistently used version 5.3 for these studies.

The gyrification pattern of the entire cortex was indexed based on the vertex-wise LGI value, a three-dimensional extension of the classical two-dimensional gyrification index measurement (i.e., the area ratio of the outer contour and corresponding pial contour) calculated by the method of Schaer et al. (34). The computing began with the creation of an outer surface using morphological closing operation. Then, approximately 800 overlapping circular regions of interest (ROIs; radius = 25 mm) were created on the outer surface. For each of these ROIs, a corresponding region of interest was defined on the pial surface. The whole computation ended up with the creation of an individual map containing one LGI value for each point of the cortical surface (i.e., ~150,000 values per hemisphere). These LGI values were used for the vertex-wise statistical analysis (e.g., group comparisons) in which significant results were shown as clusters of vertices.

Cortical thickness measurements were obtained by calculating the shortest distance from the gray/white boundary to the gray/cerebrospinal fluid boundary at each vertex on the tessellated surface.

Each image was resampled onto the average subject (fsaverage) and then smoothed either with a 5-mm, full-width, half-maximum (FWHM) Gaussian kernel (LGI) or a 10-mm FWHM Gaussian kernel (cortical thickness) to reduce the signal to noise.

### Statistical analysis

The chi-square test or an analysis of variance (ANOVA) was performed to compare group differences in demographic and clinical data.

For the analyses of MRI measures, Sz and SzTypal groups were separately treated because they probably have different neurodevelopmental pathologies in degree and duration as suggested by substantial differences in LGI (13) and cortical thickness (14). However, they were also analyzed together because of possible common neurobiology within the Sz spectrum (15). While first-episode Sz subgroup was also examined in order to reduce confounding factors (e.g., illness chronicity and medication), this should be considered as a supplementary explorative analysis in terms of its small sample size.

The effects of birth seasons on LGI or cortical thickness was examined in each diagnostic group because of a prior hypothesis that such effects (summer- *vs.* winter-born) on brain morphology would be different between diagnostic groups, which was also supported by the interaction (uncorrected *p* < 0.01) between birth seasons and diagnosis (i.e., Sz, SzTypal, and HC) by a general linear model with age and gender as nuisance covariates (**Supplementary Figures 1 and 2**).

In each group, a general linear model using age, sex, and medication (dosage and duration) as nuisance variables was used to detect the effects of birth seasons (summer- *vs.* winter-born) with more conservative threshold. Initially, clusters were formed with a threshold of uncorrected *p* < 0.05. Then a Monte-Carlo simulation was done for correction for multiple comparisons using the AlphaSim program of the Analysis of Functional NeuroImages (35) implemented in FreeSurfer. To define significant clusters, 1,000 simulations were performed for each comparison. The significance of differences was set at corrected *p* < 0.05.

## Results

### Demographic and clinical characteristics

Birth seasons (summer- *vs.* winter-born) did not differ significantly between the groups (Chi-square = 2.02, *p* = 0.364).

**Table 1** shows the demographic and clinical characteristics of participants. Age, sex, and parental education were similar in the Sz, SzTypal, and HC groups, while personal education was longer in the HC group. The total SAPS score and daily antipsychotic use were higher in the Sz group than in the SzTypal group. Birth seasons (summer- *vs.* winter-born) did not significantly affect demographic or clinical variables (**Table 1**) even within each diagnostic group.

### Brain gyrification

**Figure 1:**
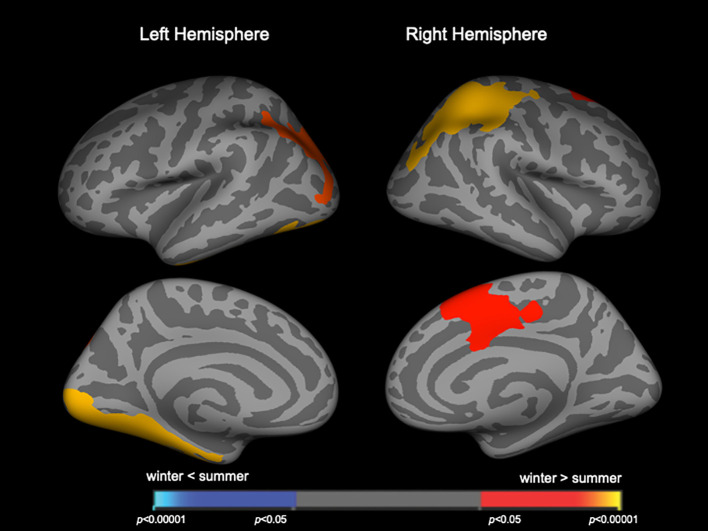
Clusters showing differences in the local gyrification index (LGI) in patients with schizophrenia. Maps are shown for the right and left hemispheres in the lateral and medial views, respectively. Horizontal bars show *p*-values corrected for multiple comparisons.

Clusters with birth season-by-diagnosis interaction were observed in the frontal and temporo-limbic regions (**Supplementary Figure 1**). While birth seasons did not significantly affect LGI in the HC or SzTypal group, LGI in the Sz group was significantly higher in winter-born patients than in summer-born patients mainly in the left lateral occipital and inferior temporal regions and right parietal and superior frontal regions (**Figure 1**, **Table 2**). A similar or enhanced effect was observed when we examined the first-episode Sz subgroup only (*n* = 64) (**Supplementary Figure 3**, **Supplementary Table 1**).

**Table 2:** Clusters with increased local gyrification index in winter-born schizophrenia patients.

Cluster no.	Cluster size (mm^2^)	Cluster-wise *p* (corrected)	MNI coordinates (maximum vertex)			Annotation
			x	y	z	
Winter-born > summer-born						
1	6505	0.0001	-14.0	-85.3	-4.2	Left cuneus cortex, pericalcarine cortex, lingual gyrus, fusiform gyrus, parahippocampal gyrus, entorhinal cortex
2	3015	0.0035	-21.2	-78.2	35.9	Left superior parietal cortex, inferior parietal cortex, lateral occipital cortex, inferior temporal gyrus
3	6108	0.0001	29.0	-41.0	59.9	Right superior frontal gyrus, precentral gyrus, postcentral gyrus, supramarginal gyrus, superior parietal cortex, inferior parietal cortex
4	2318	0.0200	10.4	13.3	47.4	Right superior frontal gyrus, caudal anterior and posterior division of the superior frontal gyrus, paracentral lobule

MNI, The Montreal Neurological Institute.

**Figure 2:**
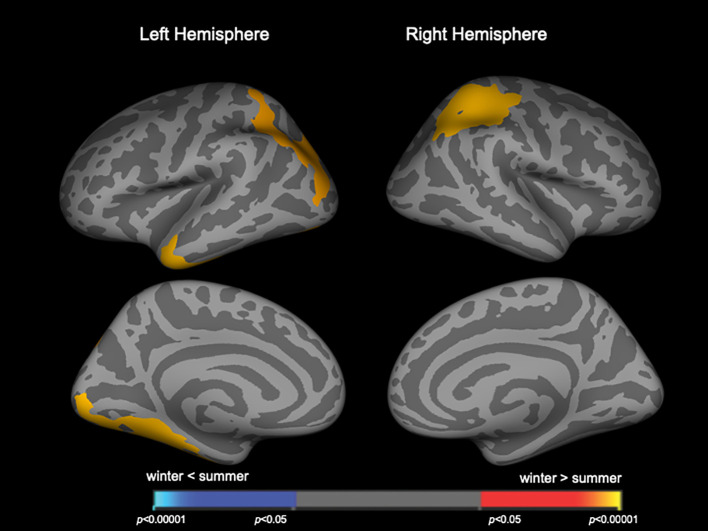
Clusters showing differences in the local gyrification index (LGI) in the combined patient group of schizophrenia and schizotypal disorder. Maps are shown for the right and left hemispheres in the lateral and medial views, respectively. Horizontal bars show *p*-values corrected for multiple comparisons.

When patient groups (Sz and SzTypal) were categorized together, winter-born patients had a higher LGI in the similar to above but smaller brain regions (**Figure 2**, **Table 3**) compared to summer-born patients. There were no clusters with a higher LGI in summer-born patients than in winter-born patients in either group.

**Table 3:** Clusters with increased local gyrification index in winter-born patients with schizophrenia and schizotypal disorder.

Cluster no.	Cluster size (mm^2^)	Cluster-wise *p* (corrected)	MNI coordinates (maximum vertex)			Annotation
			x	y	z	
Winter-born > summer-born						
1	5653	0.0000	-44.4	-14.8	-30.0	Left superior temporal gyrus, middle temporal gyrus, inferior temporal gyrus, paracalcarine cortex, lingual gyrus, fusiform gyrus, parahippocampal gyrus
2	4755	0.0001	-32.1	-74.5	29.4	Left superior parietal cortex, postcentral cortex, inferior parietal cortex, lateral occipital cortex
3	4173	0.0001	29.8	-42.0	61.1	Right inferior parietal cortex, superior parietal cortex, postcentral gyrus

MNI, The Montreal Neurological Institute.

### Cortical thickness

**Figure 3:**
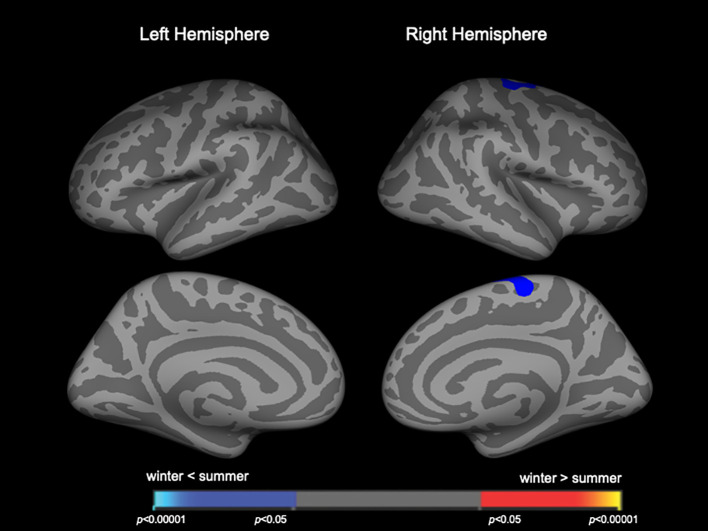
Clusters showing differences in cortical thickness in the combined patient group of schizophrenia and schizotypal disorder. Maps are shown for the right and left hemispheres in the lateral and medial views, respectively. Horizontal bars show *p*-values corrected for multiple comparisons.

Clusters with birth season-by-diagnosis interaction were observed in the widespread cortical regions (**Supplementary Figure 2**), but birth seasons did not significantly affect cortical thickness when each diagnostic group was assessed separately. When the combined patient group (Sz and SzTypal) was examined, cortical thickness in the right superior frontal region was significantly less in winter-born patients than in summer-born patients (**Figure 3**, **Table 4**). Cortical thickness in the HC group was not significantly affected by birth seasons.

**Table 4:** Clusters with reduced cortical thickness in winter-born patients with schizophrenia and schizotypal disorder.

Cluster no.	Cluster size (mm^2^)	Cluster-wise *p* (corrected)	MNI coordinates (maximum vertex)			Annotation
			x	y	z	
Summer-born > winter-born						
1	885.3	0.0383	24.1	-16.1	64.5	Right superior frontal gyrus, precentral gyrus, paracentral lobule

MNI, The Montreal Neurological Institute.

## Discussion

To the best of our knowledge, this is the first MRI study using whole-brain surface-based morphometry that revealed significant relationships between brain morphological changes and birth seasons in patients with Sz and SzTypal disorder. Winter-born patients with Sz, particularly those in the first-episode subgroup, were characterized by a higher LGI in diverse cortical regions than that in summer-born patients with Sz. This effect of birth seasons on gyrification remained significant when patient groups (Sz and SzTypal) were categorized together as a whole Sz spectrum group. We found no significant effect of birth seasons on cortical thickness in the Sz or SzTypal group; however, the right superior frontal region was thinner in winter-born patients than in summer-born patients in the whole Sz spectrum group. Therefore, the present results suggest that the brain morphological characteristics of the Sz spectrum, particularly the widespread hyper-gyrification pattern, are more evident in winter-born than in summer-born patients, which supports the role of the disruption of neural development associated with birth seasons on the pathophysiology of the Sz spectrum.

The present whole-brain cortical surface analysis supports our previous hypothesis that winter-born patients with Sz spectrum disorders show greater brain changes, particularly increased brain gyrification, than summer-born patients. The present structural MRI study cannot address the mechanisms underlying the relationship between winter-born and brain hyper-gyrification in our sample; nevertheless, evidence from immunological (36, 37) and animal (38) studies have suggested that prenatal environmental factors associated with winter, such as maternal vitamin D deficiency (5) or infection (e.g., influenza, toxoplasmosis, and meningococcal disease) (3), may disrupt synapse formation and interneuron migration during prenatal neurodevelopment due to inflammatory and immune reactions (4, 6, 39). Neuroimaging evidence has also supported the relationship between brain dysconnectivity and hyper-gyrification in Sz, which may underlie various clinical symptoms and cognitive performance of the patients (10).

In the present study, the effects of seasonality on brain gyrification were more pronounced in Sz than in SzTypal disorder, a milder form within the Sz spectrum (12), supporting greater and/or more prolonged neurodevelopmental deviations potentially leading to the overt and sustained psychosis observed in full-blown Sz (40). We have previously reported in the current sample (13) that both Sz and SzTypal patients exhibited a significantly higher LGI in diverse cortical regions, particularly in the frontal and temporo-limbic regions as well as parietal cortex, as compared with controls, but its extent was broader in Sz especially for the right prefrontal and left occipital regions. The brain regions with hyper-gyrification in winter-born patients(**Figures 1**, **2**) were largely overlapping with those brain regions. However, the lack of a significant effect of birth seasonality on prefrontal gyrification, which was previously shown to be markedly increased in Sz spectrum patients (13) and related to executive dysfunction in first-episode Sz (41), may reflect regional specificity in the trajectories of gyral formation; cortical folding predominantly occurs during gestation (17), whereas brain surface morphology may be partly affected by a late brain maturation process during adolescence particularly in the frontal region (42), which is considered to be changed in Sz (43–45) but may be largely unaffected by the birth season.

In contrast to the present results supporting epidemiological findings that link winter-born and the risk of Sz, the majority of MRI studies failed to detect a significant effect of winter-born on brain changes characteristic to Sz. As reviewed by Torrey et al. (3), earlier results of the relationship between birth seasons and ventricular sizes in Sz have been decidedly contradictory. In contrast to the findings of animal studies showing that pre- and perinatal inflammation induced changes in the histology of the hippocampus (46) or the maldevelopment of white matter (47), human MRI studies revealed the contribution of ‘summer’ births to a smaller hippocampal volume in a combined sample of Sz, depression, and HC subjects (7) or to the widespread disruption of the integrity of white matter in Sz (20). Further, our previous study using a partially overlapping Sz spectrum sample (8) identified insular hypergyria, which is a common brain characteristic of the Sz spectrum (23), in summer-born subjects irrespective of their diagnosis (i.e., Sz, SzTypal, and HC). Although these MRI findings appear to reflect environmental risk factors associated with winter in the early gestation stages in summer-born subjects, this hypothesis does not explain the established epidemiological findings of the increased risk of Sz in winter-born subjects. On the other hand, maternal infection during the second trimester, but not the first or third trimester, was also shown to increase the risk of Sz (48). Therefore, the actual timing of seasonal factors on brain development associated with the risk of Sz remains unclear (3) and warrants further study in a more integrated manner.

As predicted, birth seasons did not significantly affect cortical thickness in the Sz or SzTypal group; we only found a small cluster in the right superior frontal region where winter-born patients had thinner gray matter than summer-born patients when both patient groups were combined. Based on cortical thinning in diverse fronto-temporal regions in our Sz and SzTypal patients relative to HC subjects (14), the present results indicate that cortical thickness is not a useful measure for assessing the potential role of early environmental factors associated with birth seasonality in the pathophysiology of the Sz spectrum. This may be due to various factors that affect gray matter volume/thickness after birth, such as active reductions in gray matter around the onset of psychosis in Sz (15).

There are potential confounding factors that need to be considered. While brain gyrification is considered to better reflect early neurodevelopment than cortical thickness/volume as a stable biomarker of psychosis (44, 45), it is not entirely unaffected by disease chronicity or antipsychotic medication (49). Since the present patient groups had received antipsychotic medication for substantial periods (**Table 1**), even the first-episode Sz subgroup [mean dose = 10.3 ± 8.8 mg/day (haloperidol equivalent), duration = 7.7 ± 12.2 months], further studies on patients with antipsychotic-naïve and/or in earlier illness stages (e.g., clinical high risk for psychosis) are required to reduce these confounding factors (50, 51). Furthermore, although the present study screened for severe obstetric complications (e.g., fetal distress), more detailed clinical information (e.g., birth weeks, birth weight, pre-/perinatal infections, prenatal smoking exposure, and maternal vitamin D deficiency) on participants will be required to provide more detailed insights into the mechanisms responsible for the established epidemiological finding of birth seasonality in the Sz spectrum. Information on smoking habits of the study participants, which could also affect brain morphology (52), was also unavailable. It should be also noted that the HC group was not balanced for the season of birth as compared with patient groups (i.e., trend towards higher winter-born rate). This did not affect our main finding that birth seasons had a significant effect on brain morphology in the Sz spectrum, but their general role in normal brain development should be further tested in a larger and well-balanced healthy cohort. Moreover, we divided participants into the summer- and winter-born groups, as described in previous studies (7, 8, 20); however, several epidemiological studies examined the relationship between birth seasons and the risk of Sz using a more detailed classification based on the four seasons or birth months (2). However, the present study was considered underpowered to examine brain changes using such a detailed classification due to the small number of participants and the comprehensive nature of the brain assessment. In addition, birth season-by-diagnosis interaction on brain morphology was detected only at a relatively liberal threshold of uncorrected *p* < 0.01, which may be also due to the small sample size. Finally, because the relationship between birth seasonality and the risk of illness has also been reported in other neuropsychiatric disorders (e.g., bipolar disorder) (1, 53), the disease specificity of the present results needs to be tested in future.

In conclusion, the present MRI study expanded our previous findings on the Sz spectrum in demonstrating that seasonal birth significantly contributed to brain morphological changes in the patient group, particularly increased gyrification in diverse cortical regions (13). The present results appear to provide support for the relationship between winter-born and the increased risk of the Sz spectrum (2, 3), where the disruption of neural development may occur due to environmental factors associated with seasonality *in utero*. Further studies on a larger transdiagnostic sample in combination with more detailed clinical data (e.g., obstetric complications and maternal viral infection) and immune biomarkers are needed to clarify the relationship between pre-/perinatal environmental factors and the risk of neuropsychiatric disorders.

## Data availability statement

The raw data supporting the conclusions of this article will be made available by the authors, without undue reservation.

## Ethics statement

The studies involving humans were approved by Committee of Medical Ethics of the University of Toyama. The studies were conducted in accordance with the local legislation and institutional requirements. Written informed consent for participation in this study was provided by the participants’ legal guardians/next of kin.

## Author contributions

MT: Formal Analysis, Investigation, Methodology, Resources, Writing – original draft, Writing – review & editing. TT: Conceptualization, Funding acquisition, Investigation, Methodology, Resources, Supervision, Writing – review & editing. YT: Formal Analysis, Investigation, Methodology, Resources, Supervision, Validation, Writing – review & editing. DS: Formal Analysis, Funding acquisition, Methodology, Resources, Supervision, Validation, Writing – review & editing. HK: Resources, Writing – review & editing. KS: Resources, Writing – review & editing. YY: Resources, Writing – review & editing. NT: Funding acquisition, Writing – review & editing. KN: Methodology, Supervision, Writing – review & editing. MS: Conceptualization, Funding acquisition, Supervision, Writing – review & editing.

## References

[1] Castrogiovanni P Iapichino S Pacchierotti C Pieraccini F. Season of birth in psychiatry. A review. Neuropsychobiology. (1998) 37:175–81. doi: 10.1159/000026499 9648124

[2] Coury SM Lombroso A Avila-Quintero VJ Taylor JH Flores JM Szejko N. Systematic review and meta-analysis: Season of birth and schizophrenia risk. Schizophr Res. (2023) 252:244–52. doi: 10.1016/j.schres.2022.12.016 36682315

[3] Torrey EF Miller J Rawlings R Yolken RH. Seasonality of births in schizophrenia and bipolar disorder: a review of the literature. Schizophr Res. (1997) 28:1–38. doi: 10.1016/s0920-9964(97)00092-3 9428062

[4] Allswede DM Cannon TD. Prenatal inflammation and risk for schizophrenia: A role for immune proteins in neurodevelopment. Dev Psychopathol. (2018) 30:1157–78. doi: 10.1017/S0954579418000317 30068405

[5] Chiang M Natarajan R Fan X. Vitamin D in schizophrenia: a clinical review. Evid Based Ment Health. (2016) 19:6–9. doi: 10.1136/eb-2015-102117 26767392 PMC10699337

[6] Cheslack-Postava K Brown AS. Prenatal infection and schizophrenia: A decade of further progress. Schizophr Res. (2022) 247:7–15. doi: 10.1016/j.schres.2021.05.014 34016508 PMC8595430

[7] Schaub N Ammann N Conring F Müller T Federspiel A Wiest R. Effect of season of birth on hippocampus volume in a transdiagnostic sample of patients with depression and schizophrenia. Front Hum Neurosci. (2022) 16:877461. doi: 10.3389/fnhum.2022.877461 35769255 PMC9234120

[8] Takahashi T Sasabayashi D Takayanagi Y Kobayashi H Torigoe M Sakamoto K. Birth season and gross brain morphology associated with early neurodevelopment in schizophrenia spectrum patients and healthy subjects. Psychiatry Res Neuroimaging. (2023) 335:111714. doi: 10.1016/j.pscychresns.2023.111714 37690160

[9] Matsuda Y Ohi K. Cortical gyrification in schizophrenia: current perspectives. Neuropsychiatr Dis Treat. (2018) 14:1861–9. doi: 10.2147/NDT.S145273 PMC6055839 30050300

[10] Sasabayashi D Takahashi T Takayanagi Y Suzuki M. Anomalous brain gyrification patterns in major psychiatric disorders: a systematic review and transdiagnostic integration. Transl Psychiatry. (2021) 11:176. doi: 10.1038/s41398-021-01297-8 33731700 PMC7969935

[11] Siever LJ Kalus OF Keefe RS. The boundaries of schizophrenia. Psychiatr Clin North Am. (1993) 16:217–44. doi: 10.1016/S0193-953X(18)30171-0 8332562

[12] Siever L Davis KL. The pathophysiology of schizophrenia disorders: perspectives from the spectrum. Am J Psychiatry. (2004) 161:398–413. doi: 10.1176/appi.ajp.161.3.398 14992962

[13] Sasabayashi D Takayanagi Y Takahashi T Nemoto K Furuichi A Kido M. Increased brain gyrification in the schizophrenia spectrum. Psychiatry Clin Neurosci. (2020) 74:70–6. doi: 10.1111/pcn.12939 31596011

[14] Takayanagi Y Sasabayashi D Takahashi T Furuichi A Kido M Nishikawa Y. Reduced cortical thickness in schizophrenia and schizotypal disorder. Schizophr Bull. (2020) 46:387–94. doi: 10.1093/schbul/sbz051 PMC7406196 31167030

[15] Takahashi T Suzuki M. Brain morphologic changes in early stages of psychosis: Implications for clinical application and early intervention. Psychiatry Clin Neurosci. (2018) 72:556–71. doi: 10.1111/pcn.12670 29717522

[16] Armstrong E Schleicher A Omran H Curtis M Zilles K. The ontogeny of human gyrification. Cereb Cortex. (1995) 5:56–63. doi: 10.1093/cercor/5.1.56 7719130

[17] Zilles K Palomero-Gallagher N Amunts K. Development of cortical folding during evolution and ontogeny. Trends Neurosci. (2013) 36:275–84. doi: 10.1016/j.tins.2013.01.006 23415112

[18] Schmitt JE Raznahan A Liu S Neale MC. The heritability of cortical folding: evidence from the human connectome project. Cereb Cortex. (2021) 31:702–15. doi: 10.1093/cercor/bhaa254 PMC7727360 32959043

[19] Fischl B. FreeSurfer. Neuroimage. (2012) 62:774–81. doi: 10.1016/j.neuroimage.2012.01.021 PMC3685476 22248573

[20] Giezendanner S Walther S Razavi N Van Swam C Fisler MS Soravia LM. Alterations of white matter integrity related to the season of birth in schizophrenia: a DTI study. PloS One. (2013) 8:e75508. doi: 10.1371/journal.pone.0075508 24086548 PMC3785501

[21] Suzuki M Zhou SY Takahashi T Hagino H Kawasaki Y Niu L. Differential contributions of prefrontal and temporolimbic pathology to mechanisms of psychosis. Brain. (2005) 128:2109–22. doi: 10.1093/brain/awh554 15930048

[22] Takahashi T Suzuki M Zhou SY Nakamura K Tanino R Kawasaki Y. Prevalence and length of the adhesio interthalamica in schizophrenia spectrum disorders. Psychiatry Res. (2008) 164:90–4. doi: 10.1016/j.pscychresns.2008.03.001 18790617

[23] Takahashi T Sasabayashi D Takayanagi Y Furuichi A Kobayashi H Yuasa Y. Gross anatomical features of the insular cortex in schizophrenia and schizotypal personality disorder: Potential relationships with vulnerability, illness stages, and clinical subtypes. Front Psychiatry. (2022) 13:1050712. doi: 10.3389/fpsyt.2022.1050712 36465304 PMC9715601

[24] World Health Organization. The ICD-10 classification of mental and behavioural disorders: diagnostic criteria for research. Geneva: World Health Organization (1993).

[25] Andreasen NC Flaum M Arndt S. The comprehensive assessment of symptoms and history (CASH): an instrument for assessing diagnosis and psychopathology. Arch Gen Psychiatry. (1992) 49:615–23. doi: 10.1001/archpsyc.1992.01820080023004 1637251

[26] Andreasen NC. Scale for the assessment of negative symptoms/scale for the assessment of positive symptoms. Iowa City: The University of Iowa (1984).

[27] First MB Gibbon M Spitzer RL Williams JBW. Structured clinical interview for DSM-IV Axis I disorders. Washington DC: American Psychiatric Press (1997).

[28] Hirayasu Y McCarley RW Salisbury DF Tanaka S Kwon JS Frumin M. Planum temporale and Heschl gyrus volume reduction in schizophrenia: a magnetic resonance imaging study of first-episode patients. Arch Gen Psychiatry. (2000) 57:692–9. doi: 10.1001/archpsyc.57.7.692 PMC2850271 10891040

[29] Schooler N Rabinowitz J Davidson M Emsley R Harvey PD Kopala L. Risperidone and haloperidol in first-episode psychosis: a long-term randomized trial. Am J Psychiatry. (2005) 162:947–53. doi: 10.1176/appi.ajp.162.5.947 15863797

[30] Takahashi T Sasabayashi D Takayanagi Y Furuichi A Kobayashi H Yuasa Y. Gross anatomical variations of the insular cortex in first-episode schizophrenia. Schizophr Res. (2023) 260:23–9. doi: 10.1016/j.schres.2023.07.032 37549494

[31] Takahashi T Suzuki M Tsunoda M Kawamura Y Takahashi N Maeno N. The association of genotypic combination of the DRD3 and BDNF polymorphisms on the adhesio interthalamica and medial temporal lobe structures. Prog Neuropsychopharmacol Biol Psychiatry. (2008) 32:1236–42. doi: 10.1016/j.pnpbp.2008.03.014 18472202

[32] Filip P Bednarik P Eberly LE Moheet A Svatkova A Grohn H. Different FreeSurfer versions might generate different statistical outcomes in case-control comparison studies. Neuroradiology. (2022) 64:765–73. doi: 10.1007/s00234-021-02862-0 PMC8916973 34988592

[33] Haddad E Pizzagalli F Zhu AH Bhatt RR Islam T Ba Gari I. Multisite test-retest reliability and compatibility of brain metrics derived from FreeSurfer versions 7.1, 6.0, and 5.3. Hum Brain Mapp. (2023) 44:1515–32. doi: 10.1002/hbm.26147 PMC9921222 36437735

[34] Schaer M Cuadra MB Tamarit L Lazeyras F Eliez S Thiran JP. A surface-based approach to quantify local cortical gyrification. IEEE Trans Med Imaging. (2008) 27:161–70. doi: 10.1109/TMI.2007.903576 18334438

[35] Hagler DJ Jr Saygin AP Sereno MI. Smoothing and cluster thresholding for cortical surface-based group analysis of fMRI data. Neuroimage. (2006) 33:1093–103. doi: 10.1016/j.neuroimage.2006.07.036 PMC1785301 17011792

[36] Ellman LM Deicken RF Vinogradov S Kremen WS Poole JH Kern DM. Structural brain alterations in schizophrenia following fetal exposure to the inflammatory cytokine interleukin-8. Schizophr Res. (2010) 121:46–54. doi: 10.1016/j.schres.2010.05.014 20553865 PMC2910151

[37] Bergink V Gibney SM Drexhage HA. Autoimmunity, inflammation, and psychosis: a search for peripheral markers. Biol Psychiatry. (2014) 75:324–31. doi: 10.1016/j.biopsych.2013.09.037 24286760

[38] Ma L Li XW Zhang SJ Yang F Zhu GM Yuan XB. Interleukin-1 beta guides the migration of cortical neurons. J Neuroinflammation. (2014) 11:114. doi: 10.1186/1742-2094-11-114 24950657 PMC4084576

[39] Gumusoglu SB Stevens HE. Maternal inflammation and neurodevelopmental programming: A review of preclinical outcomes and implications for translational psychiatry. Biol Psychiatry. (2019) 85:107–21. doi: 10.1016/j.biopsych.2018.08.008 30318336

[40] Nishikawa Y Takahashi T Takayanagi Y Furuichi A Kido M Nakamura M. Orbitofrontal sulcogyral pattern and olfactory sulcus depth in the schizophrenia spectrum. Eur Arch Psychiatry Clin Neurosci. (2016) 266:15–23. doi: 10.1007/s00406-015-0587-z 25757375

[41] Sasabayashi D Takayanagi Y Nishiyama S Takahashi T Furuichi A Kido M. Increased frontal gyrification negatively correlates with executive function in patients with first-episode schizophrenia. Cereb Cortex. (2017) 27:2686–94. doi: 10.1093/cercor/bhw101 27095825

[42] Alemán-Gómez Y Janssen J Schnack H Balaban E Pina-Camacho L Alfaro-Almagro F. The human cerebral cortex flattens during adolescence. J Neurosci. (2013) 33:15004–10. doi: 10.1523/JNEUROSCI.1459-13.2013 PMC6618418 24048830

[43] Insel TR. Rethinking schizophrenia. Nature. (2010) 468:187–93. doi: 10.1038/nature09552 21068826

[44] Pantelis C Yücel M Wood SJ Velakoulis D Sun D Berger G. Structural brain imaging evidence for multiple pathological processes at different stages of brain development in schizophrenia. Schizophr Bull. (2005) 31:672–96. doi: 10.1093/schbul/sbi034 16020551

[45] Pantelis C Velakoulis D Wood SJ Yücel M Yung AR Phillips LJ. Neuroimaging and emerging psychotic disorders: the Melbourne ultra-high risk studies. Int Rev Psychiatry. (2007) 19:371–81. doi: 10.1080/09540260701512079 17671870

[46] Golan HM Lev V Hallak M Sorokin Y Huleihel M. Specific neurodevelopmental damage in mice offspring following maternal inflammation during pregnancy. Neuropharmacology. (2005) 48:903–17. doi: 10.1016/j.neuropharm.2004.12.023 15829260

[47] Favrais G van de Looij Y Fleiss B Ramanantsoa N Bonnin P Stoltenburg-Didinger G. Systemic inflammation disrupts the developmental program of white matter. Ann Neurol. (2011) 70:550–65. doi: 10.1002/ana.22489 21796662

[48] Saatci D van Nieuwenhuizen A Handunnetthi L. Maternal infection in gestation increases the risk of non-affective psychosis in offspring: a meta-analysis. J Psychiatr Res. (2021) 139:125–31. doi: 10.1016/j.jpsychires.2021.05.039 34058651

[49] Pham TV Sasabayashi D Takahashi T Takayanagi Y Kubota M Furuichi A. Longitudinal changes in brain gyrification in schizophrenia spectrum disorders. Front Aging Neurosci. (2021) 13:752575. doi: 10.3389/fnagi.2021.752575 35002674 PMC8739892

[50] Fusar-Poli P Smieskova R Kempton MJ Ho BC Andreasen NC Borgwardt S. Progressive brain changes in schizophrenia related to antipsychotic treatment? A meta-analysis of longitudinal MRI studies. Neurosci Biobehav Rev. (2013) 37:1680–91. doi: 10.1016/j.neubiorev.2013.06.001 PMC3964856 23769814

[51] Tomelleri L Jogia J Perlini C Bellani M Ferro A Rambaldelli G. Brain structural changes associated with chronicity and antipsychotic treatment in schizophrenia. Eur Neuropsychopharmacol. (2009) 19:835–40. doi: 10.1016/j.euroneuro.2009.07.007 19717283

[52] Karama S Ducharme S Corley J Chouinard-Decorte F Starr JM Wardlaw JM. Cigarette smoking and thinning of the brain’s cortex. Mol Psychiatry. (2015) 20:778–85. doi: 10.1038/mp.2014.187 PMC4430302 25666755

[53] Hsu CW Tseng PT Tu YK Lin PY Hung CF Liang CS. Month of birth and mental disorders: A population-based study and validation using global meta-analysis. Acta Psychiatr Scand. (2021) 144:153–67. doi: 10.1111/acps.13313 PMC8360113 33930177

