# Supplementary material for: Increased brain gyrification and cortical thinning in winter-born patients with schizophrenia spectrum
Source: Front Psychiatry. 2024 Apr 24;15:1368681. doi: 10.3389/fpsyt.2024.1368681 (PMC11076817; doi:10.3389/fpsyt.2024.1368681)
Supplement: Supplementary file 1 [file DataSheet_1.docx]

Supplementary Material


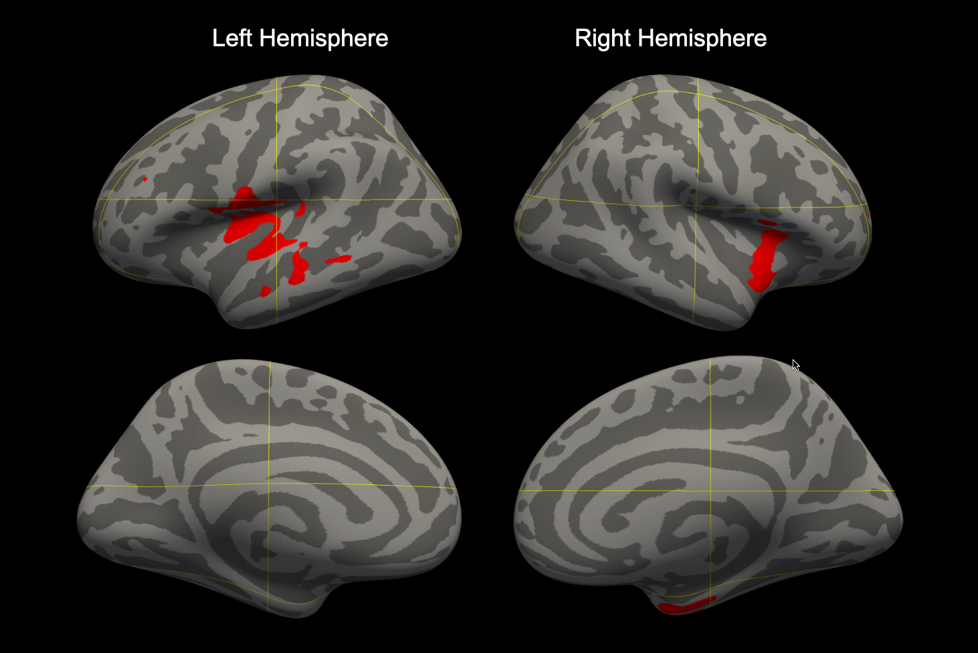


**Supplementary Figure 1.** Clusters showing birth season (summer- *vs.* winter-born)-by-diagnosis (schizophrenia, schizotypal, and healthy groups) interaction in the local gyrification index (uncorrected *p* < 0.01). Maps are shown for the right and left hemispheres in the lateral and medial views, respectively.


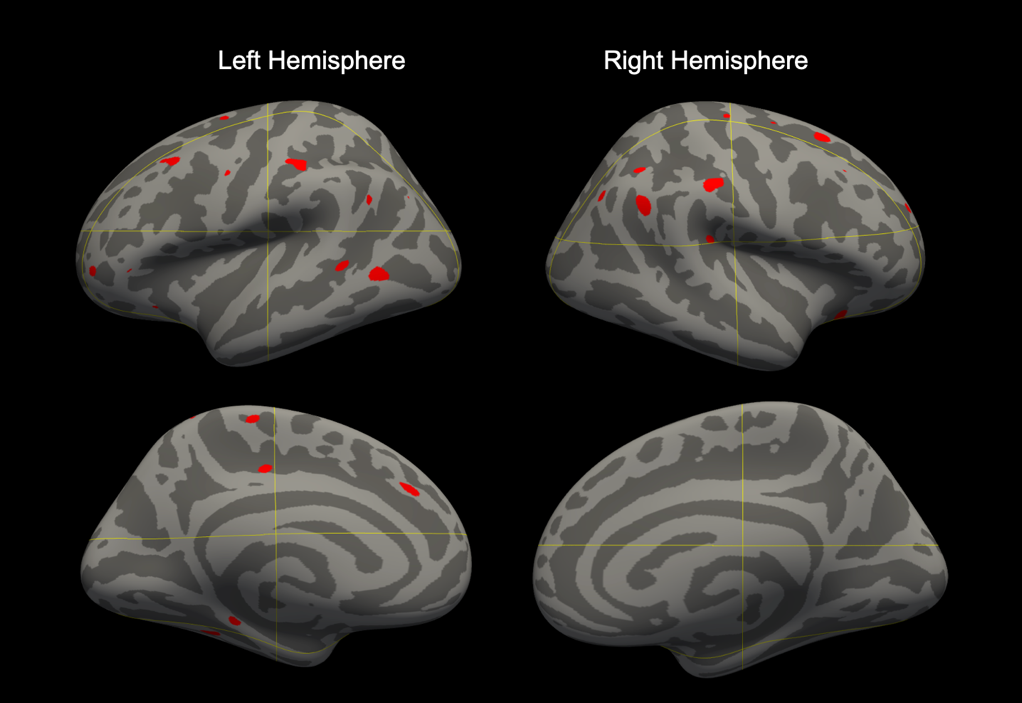


**Supplementary Figure 2.** Clusters showing birth season (summer- *vs.* winter-born)-by-diagnosis (schizophrenia, schizotypal, and healthy groups) interaction in the cortical thickness (uncorrected *p* < 0.01). Maps are shown for the right and left hemispheres in the lateral and medial views, respectively.


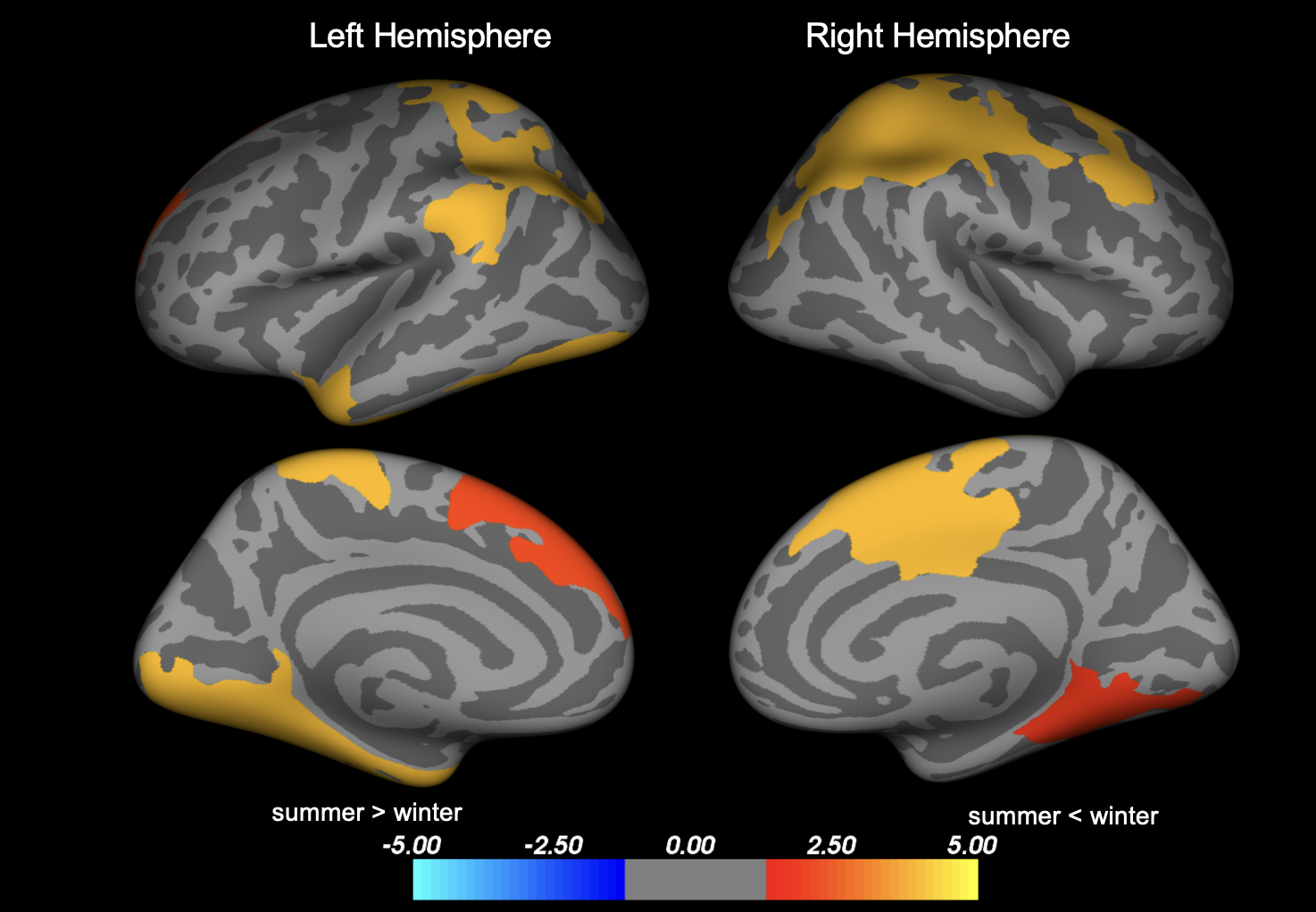


**Supplementary Figure 3.** Clusters showing differences in the local gyrification index in first-episode patients with schizophrenia (illness duration ≤ 1 year, *n* = 64). Maps are shown for the right and left hemispheres in the lateral and medial views, respectively. Horizontal bars show *p*-values corrected for multiple comparisons.
